# Increasing the Production of Volatile Fatty Acids from Corn Stover Using Bioaugmentation of a Mixed Rumen Culture with Homoacetogenic Bacteria

**DOI:** 10.3390/microorganisms9020337

**Published:** 2021-02-08

**Authors:** Nanditha Murali, Keerthi Srinivas, Birgitte K. Ahring

**Affiliations:** 1 Department of Chemical Engineering, Voiland College of Engineering and Architecture, Washington State University, Pullman, WA 99163, USA; nanditha.murali@wsu.edu (N.M.); keerthivasan1@gmail.com (K.S.); 2 Bio-Products, Sciences and Engineering Laboratory, Washington State University, Richland, WA 99354, USA; 3 Department of Biological Systems Engineering, Washington State University, Pullman, WA 99163, USA

**Keywords:** acetic acid, *Acetobacterium woodii*, bioaugmentation, homoacetogens, rumen

## Abstract

Volatile fatty acids (VFA) are industrially versatile chemicals and have a major market. Although currently produced from petrochemicals, chemical industries are moving towards more bio-based VFA produced from abundant, cheap and renewable sources such as lignocellulosic biomass. In this study, we examined the effect of bioaugmentation with homoacetogenic bacteria for increasing VFA production in lignocellulose fermentation process. The central hypothesis of this study was that inhibition of methanogenesis in an in vitro rumen bioreactor fed with lignocellulosic biomass hydrolysate increases the hydrogen partial pressure, which can be redirected towards increased VFA production, particularly acetic acid, through targeted bioaugmentation with known homoacetogenic bacteria. In this study, methanogenesis during ruminal fermentation of wet exploded corn stover was initially inhibited with 10 mM of 2-bromoethanesulfonate (BES), followed by bioaugmentation with either *Acetitomaculum ruminis* and *Acetobacterium woodii* in two separate bioreactors. During the inhibition phase, we found that addition of BES decreased the acetic acid yield by 24%, while increasing headspace hydrogen from 1% to 60%. After bioaugmentation, the headspace hydrogen was consumed in both bioreactors and the concentration of acetic acids increased 45% when *A. ruminis* was added and 70% with *A. woodii* added. This paper demonstrates that mixed microbial fermentation can be manipulated to increase VFA production through bioaugmentation.

## 1. Introduction

The chemical industry is slowly transitioning from using fossil fuels to using renewables biomass feedstocks to lower greenhouse gas emissions and their carbon footprint [1]. Although this seems like an obvious development, various obstacles must be overcome to achieve an efficient process for converting biomass to a high-value product [2]. One such obstacle is making the biomass susceptible to enzymatic hydrolysis. Lignocelluloses are naturally recalcitrant to enzymatic hydrolysis in lieu of the lignin sheath encompassing the cellulose and hemicellulose matrix [3]. Hence, pretreatment of biomass is the key to breaking down the lignin sheath efficiently. Although various pretreatment strategies including chemical pretreatment and hydrothermal pretreatment including steam explosion have been researched over the years, our laboratory has demonstrated the benefits of using the wet explosion (WEx) pretreatment [4,5]. WEx is done at an elevated temperature and pressure using only oxygen and water [6]. WEx was found to efficiently release cellulose and hemicellulose from lignocellulosic materials including straw and wood. Unlike conventional pretreatment methods, this method uses no harsh chemicals that can be detrimental to microbes during fermentation. Only low concentrations of inhibitory degradation products like furfural and hydroxymethylfurfural are produced during WEx, and the pretreated material is ready for further processing by enzymes and/or microbes [7].

Volatile fatty acids (VFA) are intermediates of anaerobic digestion produced during acidogenesis [8]. VFA are also the key fermentation product during ruminal fermentation, formed by conversion of organic feed materials by a mixed microbial consortia inhabiting the rumen. In contrast to the traditional sugar platform, where the pretreated material needs to be hydrolyzed by cellulolytic enzymes before fermentation to the desired product, the VFA platform can use pretreated biomass directly as raw material [9]. Besides, the VFA platform offers substrate flexibility, non-aseptic operations and mixed culture stability [10]. Microbes from the rumen can be used for biomass fermentation to VFA after methanogenesis has been inhibited or eliminated, a stage which has been defined as arrested anaerobic digestion [11]. The advantages of using a mixed microbial consortium such as a rumen culture during production of VFA compared to pure cultures is that there is no need for sterile conditions and no requirement for expensive enzymatic cocktails to produce sugars from pretreated biomass. However, low yields and high production costs hinder the commercial production of bio-based chemicals and fuels. Acetic acid, propionic acid, and butyric acid, in the molar ratio ~6:2:1 [10], are the main products of rumen fermentation of cellulosic substrates. Studies have found that fermentation of alkali-treated corn stover under mesophilic conditions by a rumen culture, produced a VFA yield of 0.59–0.71 g/gVS converted. The study also found that in addition to acetic, propionic, and butyric acids, small amounts of isobutyric and valeric acids were further produced [12].

The rumen primarily contains hydrogenotrophic methanogens, which use hydrogen as the electron donor to reduce carbon dioxide to methane [13]. Rumen bacteria are broadly classified into fermentative bacteria like *Fibrobacter* and *Ruminococcus*, acidogenic bacteria like *Prevotella* and *Clostridium*, acetogenic bacteria like *Acetitomaculum* and methanogenic Archaea like *Methanobrevibacter* and *Methanosarcina* [14,15]. Methanogens in the rumen work synergistically with other groups of microbes to keep the overall fermentation in balance, resulting in the production of methane and carbon dioxide besides VFA, which is adsorbed by the animal into the blood stream. It has been estimated that ruminal methanogenesis accounts for 10% energy loss in cattle [16]. Inhibition of methanogenesis in an artificial rumen bioreactor has been studied extensively, mainly to avoid energy loss in the animal [17]. These studies have indicated that an increase in H_2_ partial pressure is also, to a certain extent, inhibitory for the overall conversion in the rumen [18]. Mitigation strategies for methane has been studied using chemical inhibitors, methane analogs or ionophores. 2-Bromoethanesulfonic acid (BES), a coenzyme-M analog, will selectively inhibit methanogenesis and stimulate reductive acetogenesis [19]. In the same study, the addition of 0.03 M BES and addition of *Peptostreptococcus* products yielded 2.14 mM of acetic acid after 24 h of incubation with a mixture of hydrogen (80%) and -carbon dioxide (20%) in the headspace [19]. These studies showed no specifical inhibition of acetogenesis after BES addition to the mixed culture. However, inhibitors such as chloroform not only inhibit methanogenesis but also have an adverse effect on the H_2_-dependent acetogenesis [20].

It has been well established that reductive homoacetogenesis could be a beneficial substitution for methanogenesis, to reduce the significant energy loss to the ruminant [21]. Homoacetogens, including *Moorella thermoacetica*, a thermophilic bacteria utilizing hydrogen and carbon dioxide to autotrophically produce acetic acid, use the Wood Ljungdahl Pathway (WLP) to selectively produce acetic acid [22]. Herrero and Stuckey [23] defined bioaugmentation as “the process of adding selected strains/mixed cultures” to existing bioreactors for the improvement of fermentation efficiency. Although bioaugmentation has been studied for years in wastewater treatment and soil remediation [24], it has not been implemented successfully as it is considered less predictable than conventional bioremediation methods [23]. By the definition provided above, bioaugmentation depends on the ability of the added strain or mixed culture to work synergistically with the existing culture to perform the desired process change. Bioaugmentation of rumen fermentation with homoacetogens has been shown to be unsuccessful in the presence of living methanogens since they have a much higher affinity (10–100 times higher) for H_2_/CO_2_ when compared to the homoacetogens [25]. Bioaugmentation with homoacetogens for primarily increasing VFA production has not been previously studied. Most studies done in anaerobic digesters (AD) [26] have focused on adding specific cellulose-degradation bacteria to increase the biomethane yield [27,28]. Studies have shown that bioaugmentation of AD with thermophilic bacterial strains increased methane yield by 22–24% [29]. Other studies have shown similar trends, whereby bioaugmenting the first step of a two-step AD with the thermophilic *Caldicellulosiruptor dictyoglomus* led to a 93% increase in methane yield after 18 days of AD [28].

We hypothesized that VFA production (especially acetic acid) can be increased if methanogenesis is selectively inhibited in a rumen bioreactor, and the bioreactor is bioaugmented with H_2_-dependant homoacetogens. In the present study, we used pretreated (wet-exploded) corn stover as feedstock and a stable rumen culture grown on this substrate for three retention times. After inhibition of the methanogens by addition of BES, we further added homoacetogenic bacteria such as *Acetobacterium woodii* ATCC 29683 and *Acetitomaculum ruminis* ATCC 4738 to test the synergistic effect of the mixed microbial culture in the anaerobic digestion of lignocellulosic biomass on increasing VFA production.

## 2. Materials and Methods

### 2.1. Inoculum

Rumen fluid was collected and stored as described by Murali et al. [30]. The fresh rumen fluid was degassed under 80% N_2_: 20% CO_2_ (Oxarc^®^ Inc., Pasco, WA, USA) for 30 min, sealed and stored at –20 °C and 10% rumen fluid was added as inoculum to each of the bioreactors.

### 2.2. Substrate

Raw corn stover was obtained from Iowa State University and was processed as mentioned in our earlier study [30]. The milled corn stover was pretreated (wet exploded) as previously described by Biswas et al. [5]. The composition of the feed is shown in Table 1.

### 2.3. Bacterial Strains

Two bacterial strains, *Acetobacterium woodii* ATCC 29683 and *Acetitomaculum ruminis* ATCC 47386 were purchased from American Type Culture Collection (ATCC; Manassas, VA, USA). Both mixed bacterial and pure culture inoculum were added to the fermenters at a concentration of 1 × 10^8^ cells/mL.

### 2.4. Fermentation

#### 2.4.1. Control Fermenter

One sterilized fermenter (3 L Applikon^®^ ezControl autoclavable bioreactor (Applikon Biotechnology B.V, Schiedam, The Netherlands) was set up with pretreated corn stover at 2.5% TS. The fermenter was filled with 810 mL of substrate containing pretreated corn stover (2.5% TS), corn steep liquor (2%) (Sigma Aldrich, St. Louis, MO, USA) and 90 mL of inoculum. After stable performance for two retention times, 10 mM of BES was added to the reactor to specifically inhibit the methanogenic population [31]. The fermenter was vigorously degassed under N_2_-CO_2_ (80:20 *w*/*w*) for 45 min to remove any oxygen. Sodium hydroxide (5 N) (Sigma Aldrich, MO, USA) was used initially to adjust the pH of the fermentation broth and to maintain the pH at 6.5 throughout the fermentation. The fermentation was done at 37 °C with a stirrer speed of 200 rpm and a hydraulic retention time (HRT) of 6 days. After addition of 2-bromoethanesulfonate (BES) (Sigma Aldrich, MO, USA), the fermentation was run for 5 HRTs (total 30 days) and operated in a semi-continuous mode, with an organic loading rate of 3.75 gTS/L/day. Liquid samples were collected every day for analysis.

#### 2.4.2. Bioaugmented Fermenters

Two fermenters, one with *A. woodii* and another with *A. ruminis* were set up, similar to the control fermenter with feed added (as discussed in Section 2.4.1), and 45 mL of fresh rumen fluid and 45 mL of the respective bioaugmented strains were added. It should be noted that the BES addition in control and the bioaugmented fermenters were done at the same time of the microbial cycle, based on stabilized maximum methane production during ruminal fermentation of pretreated corn stover (data not shown). These fermenters were also operated semi-continuously, with an organic loading rate of 3.75 gTS/L/day and a hydraulic retention time of 6 days, for 5 HRTs (total of 30 days). Fermentation effluent samples were collected every day for analysis.

### 2.5. Analyses

#### 2.5.1. Measurement of VFA Using High Performance Liquid Chromatography

The fermentation effluent (2 mL), from each of the fermenters, was centrifuged at 10,000 rpm for 10 min and the supernatant was analyzed using High Performance Liquid Chromatography (HPLC). The supernatant, from all fermenters, was diluted 6 times using 4 mM sulfuric acid and filtered through a 0.2 micron PFTE filter for analysis. Analysis were done as described by Murali et al., [30] using an Aminex^®^ 87H Column 250 × 4.6 mm (Bio–Rad, Hercules, CA, USA), and a Shodex RI–101 refractive index detector on the UltiMate^®^ 3000 HPLC system (Dionex, Sunnyvale, CA, USA). Sulfuric acid (4 mM) in water was used as the eluent, flowing through the 87H column at a constant flow rate of 0.6 mL/min in a constant temperature oven at 60 °C. The total analysis time of the fermentation sample was 68 min.

#### 2.5.2. Gas Analyses Using Gas Analyzer

Head space gas measurements were done daily using the Universal Mass Spectrometry Gas Analyzer, UGA-200 (Stanford Research Systems, Sunnyvale, CA, USA).

#### 2.5.3. Calculations

Acetic, propionic, butyric and valeric acids were found to be the major acids produced during fermentation and were converted to acetic acid equivalents. The total VFA concentration is calculated in acetic acid equivalents using Equation (1) as follows:

(1) $$Total\;VFA\;in\;acetic\;acid\;equivalents\;=\;(Acetic\;acid\;)+\;(Propionic\;Acid\;*\;(\frac{{TO}_{PA}}{{TO}_{AA}})\;*\;(\frac{{MW}_{AA}}{{MW}_{PA}}))\;+\;\;(Butyric\;Acid\;*\;(\frac{{TO}_{BA}}{{TO}_{AA}})\;*\;(\frac{{MW}_{AA}}{{MW}_{BA}}))\;+\;(Valeric\;Acid\;*\;(\frac{{TO}_{VA}}{{TO}_{AA}})\;*\;(\frac{{MW}_{AA}}{{MW}_{VA}}))$$

where *MW* refers to the molecular weight of acetic acid (*AA*), propionic acid (*PA*), butyric/isobutyric acid (*BA*) and valeric/isovaleric acid (*VA*), which are the prominent *VFA* produced during fermentation and *TO* refers to the theoretical amount of oxygen required to completely breakdown each of these acids to carbon dioxide.

### 2.6. Feedstock Characterization

Feedstock characterization was done by analyzing the Total Solids (TS) and Volatile Solids (VS) and the biomass composition analysis of the feed and the effluent of each reactor. TS, VS and biomass composition analysis of the feed and effluent was done as described by Murali et al., [30].

## 3. Results and Discussion

### 3.1. Effect of BES on Rumen Fermentation and VFA Production

**Figure 1:**
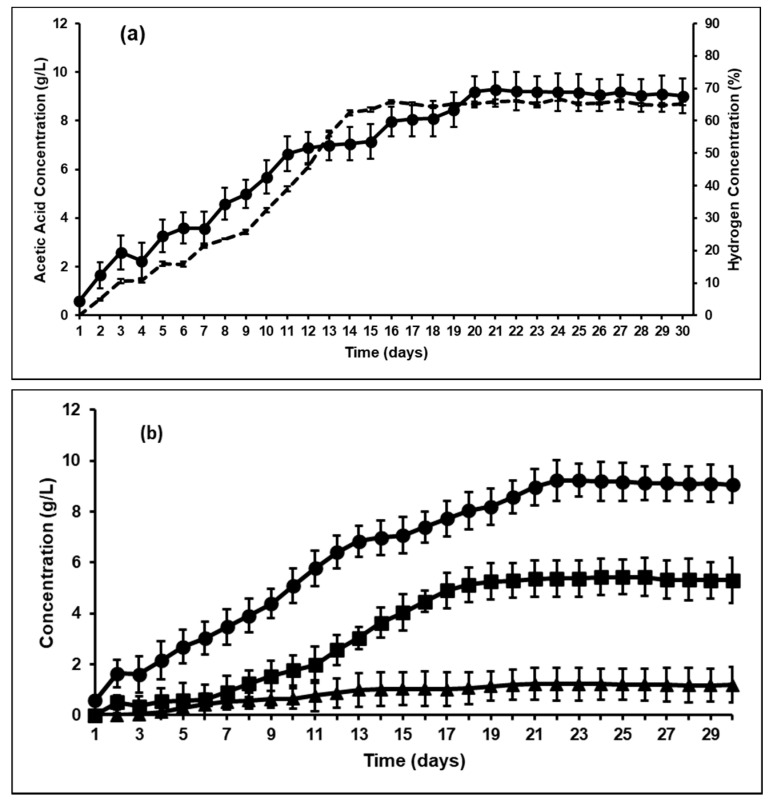
(**a**) Hydrogen concentration in headspace (wt%) versus acetic acid concentration (g/L); and (**b**) volatile fatty acids (VFA) profile; in control bioreactor after 2-bromoethanesulfonate (BES) addition and methanogenesis inhibition. (**---** Hydrogen concentration (wt%); ● Acetic acid concentration (g/L); ■ Propionic acid concentration (g/L); ▲ Butyric acid concentration (g/L)).

As indicated in Section 2.5, all the bioreactors showed stable performance for two retention times before any microbial manipulations were made and such stable performance has been previously shown [30]. Hence, in this study, day 0 is indicative of the bioreactor sample immediately after adding BES to the system and based on this assumption, the second addition of BES to the bioreactors was on day 15. This was consistently followed for both the control and the bioaugmented bioreactor. The VFA profile in the control bioreactor after BES addition is shown in Figure 1. As expected, it was found that after BES was added there was a drop in methane production to less than 5 wt% (not shown), and the hydrogen concentration in the headspace started to increase, reaching a maximum value of 60 wt% after 5 days (Figure 1a). Similar trends were observed in other studies, where inhibition of methanogens resulted in an increased hydrogen partial pressure $P_{H_{2}}P_{H_{2}}$ in the headspace [18]. Studies have shown that with complete inhibition of methanogenesis, $P_{H_{2}}$ increased from 0.087 mM/day to 1.83 mM/day in continuous cultures [32]. It can be seen from Figure 1b that acetic acid is the most prominent VFA produced, along with propionic and a low amount of butyric acid (very minute concentrations of valeric acid were produced and it was not consistent; hence, it is not shown in the graphs).

While BES was not shown to have an adverse effect on specific homoacetogens, some studies have indicated that BES can inhibit certain non-methanogenic microbial populations too [18,33]. As seen from Table 2, BES addition decreased the overall VFA yield from ruminal fermentation of pretreated corn stover from 31.09 g/L (1.25 g/gVS) to 21.41 g/L(0.95 g/gVS). The acetic acid and total VFA productivity in the bioreactor without methanogens were 1.5 g/L/day and 3.2 g/L/day, respectively, and in the bioreactor with methanogenesis [30], the acetic acid and total VFA productivity was 2 g/L/day and 5.4 g/L/day, respectively. Individual VFAs also showed a similar trend (Table 2), where acetic, propionic, and butyric acids were reduced by 24%, 44%, 49%, respectively, in reactors with BES addition compared to reactors without BES. Similar adverse effect of BES on VFA production during rumen fermentation was seen when using a pure cellulosic substrate such as Avicel [18]. Studies on rumen fermentation have shown that increased hydrogen production caused by methanogen inhibition using BES, would thermodynamically disfavor acetogenesis and instead flow [H] into other [H] sinks such as propionate, lactate, formate, succinate, etc. [34]. It can be seen from Table 2 that propionate production also dropped after BES addition and a similar effect was also found in other literature [35]. Another demonstrated effect is the adverse impact of high $P_{H_{2}}$ on the cellulose degradation, which eventually affected VFA production [18,36].

### 3.2. Effect of Bioaugmentation on Overall VFA Yield

**Figure 2:**
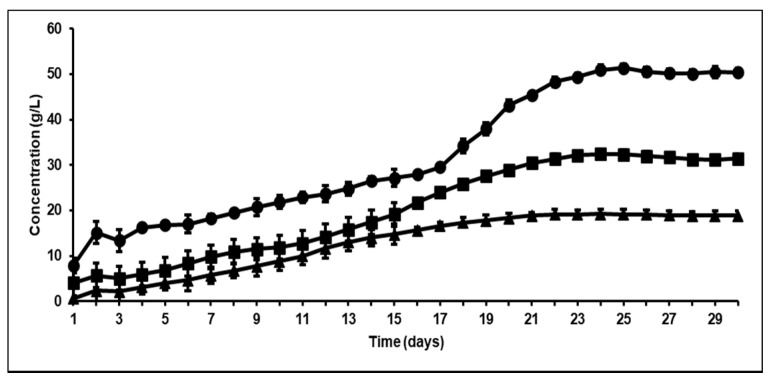
Comparison between total VFA concentration (g/L) as a function of time in control versus bioaugmented bioreactors (▲ control bioreactor without methanogenesis; ■ bioreactor augmented with *A. ruminis*; ● bioreactor augmented with *A. woodii*).

Based on the results discussed in Section 3.1, it was postulated that bioaugmentation of homoacetogens (*A. woodii* and *A. ruminis*) in fermenters that had a high hydrogen partial pressure $\left(P_{H_{2}}\right)\left(P_{H_{2}}\right)$ in the headspace would serve as an efficient alternate [H] sink, resulting in an increased acetic acid production. Previous studies have shown that such homoacetogens have shown significant growth and acetate production at such high hydrogen partial pressures with CO_2_ as the primary carbon substrate [19]. It can be seen from Figure 2 (and Table 2) that bioaugmentation with homoacetogens increased total VFA production (32.33 g/L (1.34 g/gVS) in the *A. ruminis*-augmented bioreactor and 49.31 (2.19 g/gVS) g/L in the *A. woodii*-augmented fermenter) when compared to the non-augmented control bioreactor without methanogenesis (21.41 g/L or 0.95 g/gVS). The most significant trend observed in Table 2 is that bioaugmentation with *A. ruminis* (which was primarily isolated from rumen sources) resulted in an almost similar total VFA yield as that of original rumen, further proving our hypothesis that a homoacetogen capable of growing under high $P_{H_{2}}$ can serve as an efficient [H] sink, resulting in effective cellulosic degradation to VFA.

**Figure 3:**
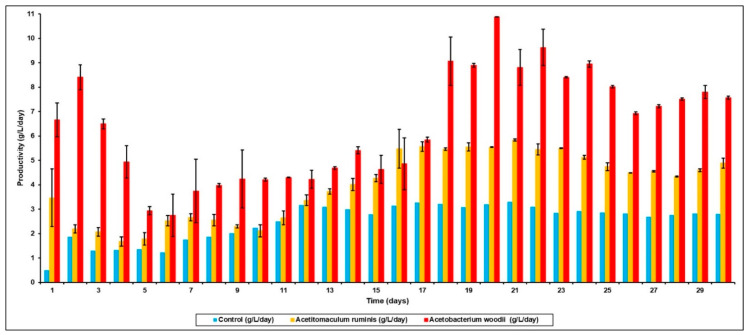
Total VFA productivity (g acetic acid equivalents/L/day) in control bioreactor after BES addition versus that bioaugmented with *A. ruminis* and *A. woodii*.

Previous studies have indicated that any changes to bioreactor performance after bioaugmentation are usually only operational for short periods of time [37]. This is primarily because of competition between the different microbial strains, and the fact that a system such as the rumen has developed over long periods of time and the resulting micro-flora is based on survival of the fittest. However, when methanogenesis is inhibited there is an opportunity for other strains to take up the space inhabited by these microbes, and as shown, this will lead to the increased production of more VFA, especially acetic acid. This effect is successfully shown in Figure 3, where the VFA productivity after bioaugmentation with *A. ruminis* or *A. woodii* was 6.2 g/L/day and 10.4 g/L/day, respectively, almost 2- to 3-fold higher than in the control reactor without bioaugmentation.

### 3.3. Effect of Bioaugmentation on Individual VFA Yield

The increase in VFA production, in particular, was in acetic (45%), propionic (18%) and butyric acids (59%) in the *A. ruminis*-augmented bioreactor, when compared to the control bioreactor (after BES addition). However, in the bioreactor augmented with *A. woodii*, the percentage increase, when compared to control, was even higher—70%, 29% and 68% of acetic, propionic and butyric acids, respectively.

**Figure 4:**
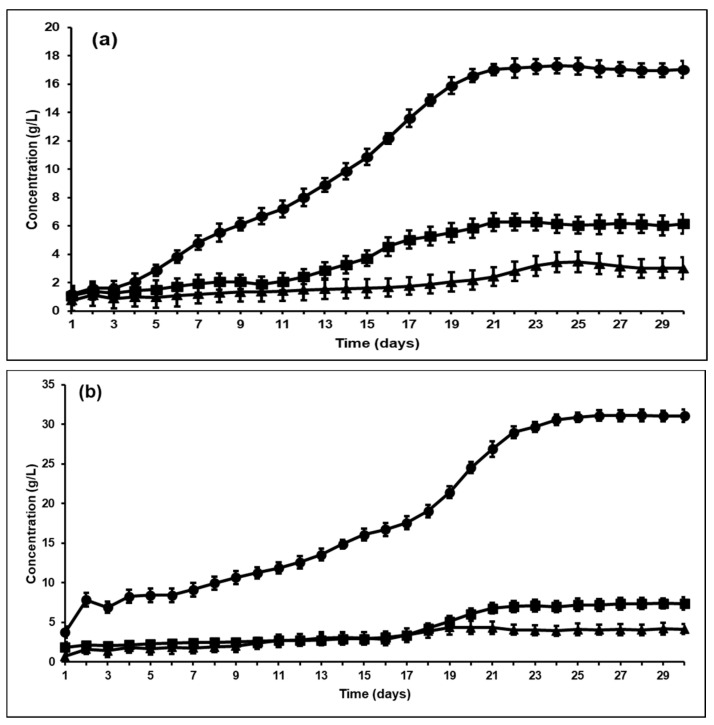
VFA (g/L) concentration as a function of time in bioreactors after BES addition and bioaugmentation with (**a**) *Acetitomaculum ruminis* and (**b**) *Acetobacterium woodii* (● Acetic acid concentration (g/L); ■ Propionic acid concentration (g/L); ▲ Butyric acid concentration (g/L)).

**Figure 5:**
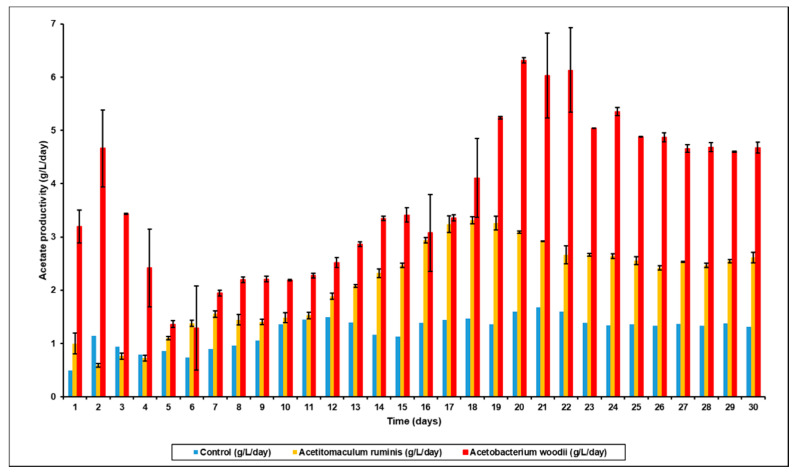
Acetate productivity (g/L/day) in control bioreactor after BES addition versus that bioaugmented with *A. ruminis* and *A. woodii*.

The variations in the VFA concentrations as a function of time in the augmented bioreactors is shown in Figure 4. With *A. ruminis* (Figure 4a), the acetic acid concentration continually increased from day 1 to day 19, after which the acetic acid production stabilized at 16.9 g/L (0.69 g/gVS) and similar trends were seen with propionic and butyric acid production. However, when bioaugmented with *A. woodii*, the acetic acid production was even higher, and the bioreactor reached stability on day 21 with 29.9 g/L (1.29 g/gVS) of acetic acid (Figure 4b). It can also be seen from Figure 5 that the acetic acid productivity in *A. ruminis* and *A. woodii* augmented bioreactors was 3.9 g/L/day and 6.3 g/L/day, respectively. It can also be seen from Figure 5 that the acetate productivity trends mirrored that of the total VFA productivity, indicating that the production of other organic acids such as propionic and butyric acid did not significantly affect the total VFA production. This further confirms that increased acetate yield from bioaugmentation with homoacetogens specifically increased acetate production by serving as an efficient [H] sink (i.e., effectively replacing the methanogens).

**Figure 6:**
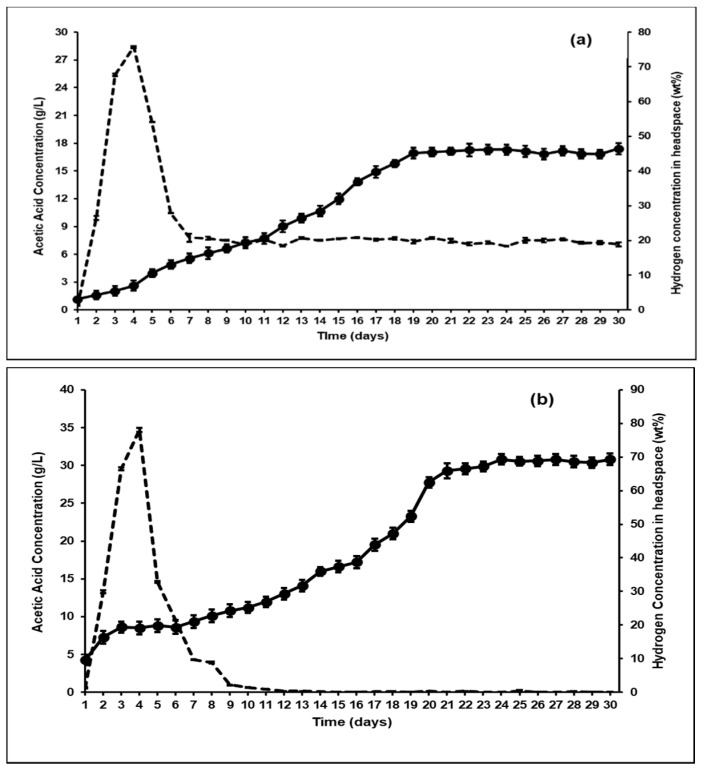
Hydrogen concentration in headspace(wt%) versus acetic acid concentration (g/L) in semicontinuous bioreactors bioaugmented with (**a**) *Acetitomaculum ruminis* and (**b**) *Acetobacterium woodii* (**---** Hydrogen concentration (wt%); ● Acetic acid concentration (g/L)).

Other studies have found similar results, where bioaugmentation with *A. woodii* induced a homoacetogenic fermentation with heat activated sludge [38]. They found that from 1 mole of glucose, almost 1.19 mole of acetic acid was produced with *A. woodii* augmentation. Previous studies have reported similar results, that inhibition of methanogenesis possibly shifts ruminal fermentation towards homoacetogenesis, especially in the presence of *A. ruminis* [21]. Lopez et al. [39] also showed that inhibition of methanogenesis increases the hydrogen scavenging ability of hydrogenotrophic homoacetogenic bacteria like *Eubacterium*, *Acetitomaculum* and *Acetobacterium*, thereby increasing acetic acid yields by almost 51%. The results from this study confirmed the hypothesis that reductive acetogenesis is indeed an alternative hydrogen sink to methanogenesis. However, while the bioaugmentation showed an increase in VFA production (especially acetic acid), the difference between the yields and productivities in *A. ruminis* and *A. woodii* augmented fermenters showed significant differences (Figure 3 and Figure 5). As seen from Figure 1b, both H_2_ and acetic acid concentrations in the control bioreactor increased after BES addition. This trend was similar to previous studies that reported that, at high partial pressure of hydrogen, homoacetogenesis can be favored [40]. These studies also indicated that methanogenesis is favored at lower H_2_ partial pressures and hence, such a significant effect of bioaugmentation on VFA production using rumen may not have been achieved without first inhibiting the methanogenic population within the rumen. It can be seen from Figure 6a,b that the H_2_ partial pressure in the fermenter headspace decreased upon bioaugmentation with both tested strains, *A. ruminis* and *A. woodii*. However, while the H_2_ concentration decreased to around 20 wt% and stayed constant as a function of time in the *A. ruminis*-augmented bioreactor, hydrogen was almost non-detectable in the *A. woodii*-augmented bioreactor after 9 days, indicating comparatively superior performance.

These differences indicate that *A. woodii* has a greater capability to completely consume the headspace hydrogen with CO_2_ to produce acetic acid through the Wood-Ljundahl pathway compared to *A. ruminis*. Some previous studies using *A. woodii* grown on H_2_/CO_2_ gaseous substrates have shown ascetic acid production of 20.37 g/L/day, with the highest concentration found amongst hydrogenotrophic acetogens [41,42]. Another reason for the comparatively ineffective performance of *A. ruminis*-augmented reactor at H_2_ concentrations below 20% could be that the H_2_ concentration was below the threshold value that is usually required by *A. ruminis* for acetic acid production. This might explain the steady state concentration of hydrogen seen in the headspace of the fermenter bioaugmented with this strain (Figure 6a).

### 3.4. Mass Balance Analysis and Feedstock/Effluent Characterization

As discussed in Section 3.3, bioaugmentation resulted in higher VFA production (especially acetic acid) indicating that reductive homoacetogenesis will be active and utilize excess H_2_ in the bioreactor headspace after inhibition of methanogenesis. While the effective utilization of H_2_ in the headspace of the ruminal fermentation directly relates to an increase in VFA production, there is an added benefit of increased cellulolytic activity in the rumen. As previously discussed, studies using BES for inhibiting methanogenic activity have also shown a decrease in the cellulolytic activity of the ruminal bacteria with an increase in the H_2_ pressure [43]. This could also be attributed to the decreased VFA production in the control bioreactor. Table 3 shows the compositional analysis done at stable bioreactor conditions (day 30) to assess the carbohydrate degradation rate in the control fermenter in comparison to the bioaugmented bioreactors. It can be seen that the cellulose and hemicellulose concentrations in the bioaugmented bioreactors were significantly lower than that in the control bioreactor. The difference in cellulose and hemicellulose (total carbohydrate) consumption in the *A. ruminis* and *A. woodii* augmented reactors were found to be 18.3 wt% and 22.7 wt%, respectively. We found no evidence of any lignin degradation, either with or without bioaugmentation. As previously found, there were no expectations on ligninolytic activity in the rumen at these conditions [30]. Assuming 1 g of carbohydrate could theoretically produce ca. 1 g of acetic acid, the carbohydrate consumption alone does not relate to the significant increase in acetic acid concentration in the effluent from *A. woodii* augmented fermenter. This difference in acetic acid concentrations between the two bioaugmented fermenters, however, could only be explained by the difference in carbohydrate consumption and increased consumption of the H_2_ in the *A. woodii* augmented fermenter (Table 3).

## 4. Conclusions

Inhibition of methanogenesis during ruminal fermentation has been well-studied, primarily, aimed at reducing methane emission from the cattle population. These studies have, however, shown that disruption of methanogenesis has a negative effect on the overall rumen fermentations and that the H_2_-scavenging function of the methanogens are important for a stable rumen function. In our study, we examined if homoacetogenes can substitute the role of methanogens during rumen fermentation and result in an increased production of VFA during fermentation of corn stover. The study is focused on two acetogenic strains, *A. ruminis* and *A. woodii*, which were both found to be capable of working in synergy with the ruminal consortia after BES addition, resulting in an increased VFA (particularly, acetic acid) production. The efficient utilization of the excess H_2_ accumulated after inhibition of methanogenesis was shown to increase the total VFA production to between 32.33 g/L (1.34 g/gVS) and 49.31 g/L (2.19 g/gVS) (acetic acid equivalents) compared to the control (i.e., non-augmented) bioreactor without methanogenesis (21.41 g/L or 0.95 g/gVS, acetic acid equivalents).

While bioaugmentation has been used previously, there has been no previous research studying the effect of bioaugmentation on improving VFA production. In another study conducted at our lab, we found that the efficient utilization of H_2_ in the bioaugmented reactors resulted in an increase in the cellulolytic activity during ruminal fermentation [18]. It was also found that an efficient H_2_-degrading acetogen, *A. woodii*, performed significantly better than *A. ruminis*, which seemed to be limited by a high H_2_ threshold concentration. The results from this current study not only increases our fundamental understanding of the effect of bioaugmentation in ruminal fermentation aimed at reducing methane activity, but also serves as an optimal alternative bioengineering tool capable of increasing the future biochemical production of organic acids.

## Figures and Tables

**Table 1:** Compositional data for pretreated (wet exploded) corn stover [30].

	Cellulose (wt%)	Hemicellulose (wt%)	Lignin (wt%)	Ash (wt%)	Total Solids (wt%)	Volatile Solids (wt%)
Pretreated Corn Stover	36.8	16.3	43.4	3.4	2.5	2.35

**Table 2:** Volatile fatty acids concentrations (g/L) in bioreactors with and without bioaugmentation and with 10 mM BES added. The bioreactors with and without methanogenesis were regarded as controls.

Bioreactor	Acetic Acid (g/L)	Propionic Acid (g/L)	Butyric Acid (g/L)	Total VFA in Acetic Acid Equivalents (g/L)
Control; With Methanogenesis [30]	12.26	10.08	2.42	31.09 (1.25 g/gVS)
Control; (BES-added) Without Methanogensis	9.29	5.63	1.23	21.41 (0.95 g/gVS)
Bioaugmentation with *A. ruminis* after BES addition	16.99	6.88	2.98	32.33 (1.34 g/gVS)
Bioaugmentation with *A. woodii* after BES addition	30.8	7.91	3.89	49.31 (2.19 g/gVS)

**Table 3:** Compositional analysis of wet exploded corn stover feed and effluent before and after bioaugmentation on day 30 of fermentation.

	Feed ^1^	Effluent A ^2^	Effluent B ^3^	Effluent C ^4^
Solid Fraction *				
Total Carbohydrates (%g/g biomass)	53.1	44.4	34.8	30.4
Cellulose (%g/g biomass)	36.8	31.9	25.9	23.6
Hemicellulose (%g/g biomass)	16.3	12.5	8.9	6.8
Soluble Lignin (%g/g biomass)	2.64	3.7	3.6	4.2
Insoluble Lignin (%g/g biomass)	40.8	40.9	50.9	54.6
Carbohydrate:Lignin Ratio	1.22	0.99	0.64	0.52
Liquid Fraction				
Acetic acid (g/L)	0.34	9.29	16.99	30.8
Propionic acid (g/L)	0.17	5.63	6.88	7.91
Butyric acid (g/L)	0.11	1.23	2.98	3.89
Hydrogen concentration (wt%)	N/A	65%	4%	0

* Solid fraction was obtained after filtration and washing with water. ^1^ Feed: Wet exploded corn stover 2.5% TS [30]. ^2^ Effluent A: Effluent on Day 30 of fermentation without methanogens (Control) [30]. ^3^ Effluent B: Effluent on Day 30 of fermentation bioaugmented with *Acetitomaculum ruminis.* ^4^ Effluent C: Effluent on Day 30 of fermentation bioaugmented with *Acetobacterium woodii.*

## Data Availability

Not Applicable.
